# Targeting breast cancer cells by MRS1477, a positive allosteric modulator of TRPV1 channels

**DOI:** 10.1371/journal.pone.0179950

**Published:** 2017-06-22

**Authors:** Mustafa Nazıroğlu, Bilal Çiğ, Walter Blum, Csaba Vizler, Andrea Buhala, Annamária Marton, Róbert Katona, Katalin Jósvay, Beat Schwaller, Zoltán Oláh, László Pecze

**Affiliations:** 1 Neuroscience Research Center, Suleyman Demirel University, Isparta, Turkey; 2 Department of Neuroscience, Health Science Institute, Suleyman Demirel University, Isparta, Turkey; 3 Unit of Anatomy, Department of Medicine, University of Fribourg, Fribourg, Switzerland; 4 Institute of Biochemistry, Biological Research Center of the Hungarian Academy of Sciences, Szeged, Hungary; 5 Institute of Chemistry, Faculty of Materials Science and Engineering, University of Miskolc, Miskolc-Egyetemváros, Hungary; 6 Acheuron Ltd., Szeged, Hungary; University of South Alabama Mitchell Cancer Institute, UNITED STATES

## Abstract

There is convincing epidemiological and experimental evidence that capsaicin, a potent natural transient receptor potential cation channel vanilloid member 1 (TRPV1) agonist, has anticancer activity. However, capsaicin cannot be given systemically in large doses, because of its induction of acute pain and neurological inflammation. MRS1477, a dihydropyridine derivative acts as a positive allosteric modulator of TRPV1, if added together with capsaicin, but is ineffective, if given alone. Addition of MRS1477 evoked Ca^2+^ signals in MCF7 breast cancer cells, but not in primary breast epithelial cells. This indicates that MCF7 cells not only express functional TRPV1 channels, but also produce endogenous TRPV1 agonists. We investigated the effects of MRS1477 and capsaicin on cell viability, caspase-3 and -9 activities and reactive oxygen species production in MCF7 cells. The fraction of apoptotic cells was increased after 3 days incubation with capsaicin (10 μM) paralleled by increased reactive oxygen species production and caspase activity. These effects were even more pronounced, when cells were incubated with MRS1477 (2 μM) either alone or together with CAPS (10 μM). Capsazepine, a TRPV1 blocker, inhibited both the effect of capsaicin and MRS1477. Whole-cell patch clamp recordings revealed that capsaicin-evoked TRPV1-mediated current density levels were increased after 3 days incubation with MRS1477 (2 μM). However, the tumor growth in MCF7 tumor-bearing immunodeficient mice was not significantly decreased after treatment with MRS1477 (10 mg/ kg body weight, i.p., injection twice a week). In conclusion, in view of a putative *in vivo* treatment with MRS1477 or similar compounds further optimization is required.

## Introduction

Malignant tumors often develop at sites of chronic tissue injury and repair, which have an important role in the pathogenesis of malignant disease, with chronic inflammation being the most important risk factor [1]. The inflammatory microenvironment contributes to tumor progression by supplying bioactive molecules, including growth factors, survival factors and extracellular matrix-modifying enzymes [2]. By creating their own inflammatory microenvironment, cancer cells increase their independency from the regulating signals from the body and block the normal healing process. In the “unfriendly” environment created by the cancer cells neither the tumor-bordering normal non-mutated epithelial cells nor the immune system may function properly [3].

The transient receptor potential cation channel (TRP) vanilloid member 1 (TRPV1) is a subfamily member of TRP channels that trigger intracellular signaling by an increase of the intracellular free Ca^2+^ concentration [Ca^2+^]_i_, Activation is triggered by multiple pain-inducing stimuli including inflammatory endovanilloids, heat (37–42^°^C), acids (pH<6.3) and pungent exovanilloids such as capsaicin (CAPS) or resiniferatoxin [4–8]. Endovanilloids are frequently produced in the “inflammatory soup”; anandamide, 12(S)-hydroxyeicosatetraenoic acid (12(*S*)-HETE), and other lipoxygenase metabolites of arachidonic and linoleic acids serve as potent agonists of TRPV1 [5,9,10]. These products are also present in the tumor microenvironment [11,12].

Expression of TRPV1 channels in neoplastic breast tissue has been reported before [13,14], but little is known about the channel’s physiological function and the likely pathological consequences in these neoplasms. It has been known for a long time -even before TRPV1 channels were discovered- that lipoxygenase products, now well-known endogenous agonists of TRPV1 channels, promote tumor growth [15]. On the other hand, virtually all pharmacological and molecular methods used to examine the function of these channels resulted in a decrease of cell viability. These methods included molecular up- or downregulation of the channels and activation or inhibition of channels with natural exogenous agonists or synthetic antagonists [13,16,17]. Strong and persistent activation of TRPV1 in sensory neurons by exogenous agonists leads to the elimination of TRPV1-expressing neurons due to a necrotic-type, overstimulation-based cytotoxicity [18–21]. However, exogenous TRPV1 agonists, although reducing tumor cell viability at high concentrations *in vitro* [13,17], fail to induce the overstimulation-based cytotoxicity observed in pain-sensing neurons, even at lower concentrations [13]. Potent natural agonist such as CAPS and RTX generally cannot be given systemically or in large doses, since it produces acute pain, neurological inflammation and a decrease in the core body temperature [22,23].

**Fig 1:**
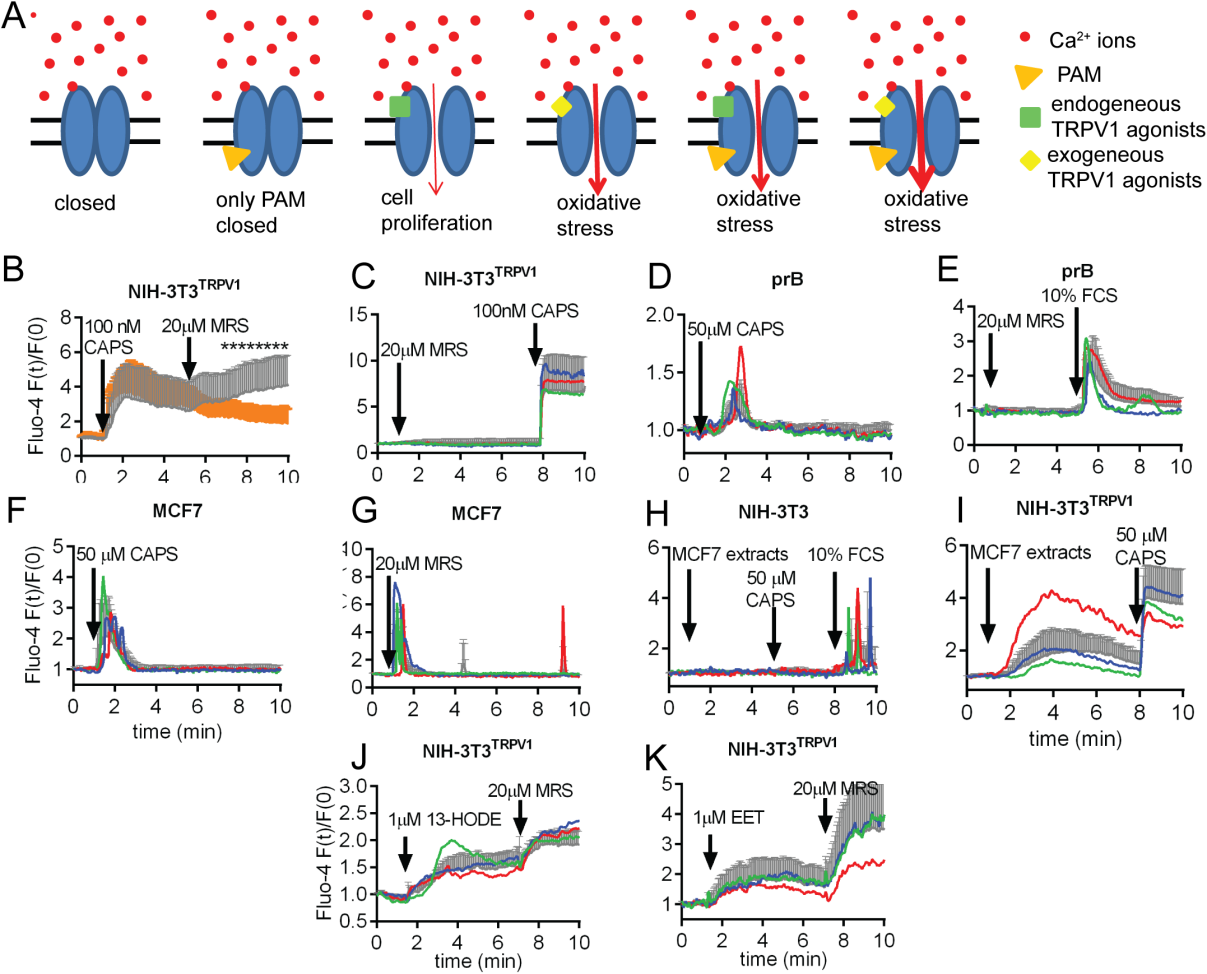
Effect of MRS on TRVPV1-mediated Ca^2+^ signaling. **A)** Schematic model of TRPV1 channel modulation by MRS in cancer cells. Homo-tetrameric TRPV1 is permeable to cations, notably Na^+^ and Ca^2+^. I) In the absence of TRPV1 agonist the channel is closed. II) Binding of a positive allosteric modulator (PAM) alone, e.g. MRS, does not activate channel opening. III) Endogenous TRPV1 agonists present in the tumor microenvironment are weak stimulators of TRPV1, possibly involved in tumor progression. IV) Exogenous agonists such as CAPS are potent TRPV1 activators. Resulting from TRPV1 hyper-activation, CAPS induces oxidative stress. V-VI) MRS amplifying the effect of both endogenous and exogenous agonists may evoke a more pronounced cytotoxic effect (oxidative stress). **B-I)** Acute effects of MRS on the intracellular Ca^2+^ regulation in various cell types. The different substances were added at the time points indicated by arrows and remained in the solution until the end of the experiments **B)** Fluorescence recordings from time-lapse videos show an increase in [Ca^2+^]_i_ after CAPS administration in NIH-3T3 cells stably transfected with the TRPV1 channel. Traces represent mean + SD. More than 20 single cell recordings were evaluated (n > 20). MRS added at t = 5 min evoked a second long-lasting elevation in [Ca^2+^]_i_. (gray curve). In cells stimulated by CAPS only, [Ca^2+^]_i_ continuously decreased after the initial peak at approximately 2 min (orange curve). Statistically significant differences between the two curves (Student t-test, p<0.05) are marked with asterisks. **C-I)** Single-cell (colored traces) and average fluorescence recordings of the entire cell population (grey traces) from time-lapse videos show changes in [Ca^2+^]_i_. Bars represent SD. All experiments were repeated two or more times with similar results. **C)** NIH-3T3^TRPV1^ cells responded to CAPS with an increase in [Ca^2+^]_i_, but not to MRS alone. **D)** Primary breast epithelial cells (prB) responded to CAPS with a brief increase in Ca^2+^]_i_ often lasting for less than a min. **E)** Primary breast epithelial cells did not respond to MRS1477 alone, but responded to serum re-administration. **F)** MCF7 breast cancer cell line responded to CAPS alone and **G)** to MRS1477 alone with brief Ca^2+^ transients. **H)** Neither MCF7 cell extracts nor CAPS (50 μM) evoked an elevation in Ca^2+^]_i_ in control (un-transfected) NIH-3T3 cells; a Ca^2+^ signal was induced by serum re-administration **I)** Application of a MCF7 cell extract increased [Ca^2+^]_i_ in NIH-3T3^TRPV1^ cells; the Ca^2+^ signal was further increased by CAPS (50 μM). **J-K**) MRS augmented the endogenous agonist-evoked rises in [Ca^2+^]_i_. The initial rise in [Ca^2+^]_i_ was evoked either by 13-HODE (1 μM) (**J**) or by 8,9-EET (1μM) (**K**).

One of the authors of this study had previously noted that MRS1477, a dihydropyridine derivative acts as a positive allosteric modulator (PAM) of TRPV1, when added together with a TRPV1 agonist. Yet MRS1477 has little or no effect on cells expressing TRPV1 either endogenously or ectopically, if added alone [24–26] (for details, see Fig 1A). MRS1477 was found I) to be a specific modulator of TRPV1 channels, in contrary to other dihydropyridine derivatives showing no allosteric effects on TRPV1 [24], II) to further increase the sensitivity of TPRV1 already sensitized with low pH (6.0) or protein kinase C phosphorylation [26] and III) to modulate the effect of endogenously produced TRPV1 agonists [25]. MRS1477 did not affect channel inhibition by capsazepine, a competitive TRPV1 antagonist, indicating a distinct MRS1477 binding site on TRPV1 for positive allosteric modulation [26].

Positive modulation of TRPV1 function(s) might lead to cytotoxicity, because excess activity of TRPV1 channels evokes not only necrotic processes with membrane bleb formation [16,20], but also apoptotic processes [27]. In addition, we reported recently that MCF7 cell proliferation is decreased by increased oxidative stress. Oxidative stress was found to be the consequence of the failure of Ca^2+^ extruding systems to cope with an excess of Ca^2+^ ions entering the cell through TRPV1 channels [28].

The evoked Ca^2+^ response highly depends on the density of TRPV1 on the plasma membrane [13]. For instance, nanomolar CAPS concentrations suffice to evoke Ca^2+^ responses in cell lines ectopically expressing TRPV1 [29,30], 0.5–1 μM CAPS is required to evoke Ca^2+^ responses in TRPV1+ sensory neurons [31], but 10–50 μM CAPS are required to evoke Ca^2+^ responses in cancer cells [13,32]; the latter endogenously expressing much lower levels of TRPV1 than TRPV1+ neurons [13]. Of note, CAPS exerts TRPV1-independent effects, however at concentrations above 250 μM [33]. CAPS-induced necrotic processes can be evoked in cells expressing high levels of receptors such as TRPV1-positive pain-sensing neurons or cells ectopically expressing TRPV1 receptor. Nevertheless, cancer cells expressing lower levels of TRPV1 proteins [13] might be still sensitive to TRPV1-mediated apoptotic processes, possibly in the presence of a TRPV1 modulator as explained in Fig 1A.

We hypothesized that the compound MRS1477 might be able to increase the oxidative stress levels in tumor cells expressing TRPV1 channels that are also producing endogenous TRPV1 agonists. Since in an otherwise healthy body TRPV1 agonists are only produced in the tumor microenvironment, PAMs of TRPV1 might have tumor-selective cytotoxic effects.

## Materials and methods

### Reagents

Capsaicin (CAPS), a TRPV1 agonist and capsazepine (CapZ), a well-characterized antagonist of TRPV1 were dissolved in DMSO at a concentration of 100 mM (all from Sigma-Aldrich, St. Louis, MO). 4-Ethyl-1,4-dihydro-5-[[(2-methoxyethyl)thio]carbonyl]-6-methyl-2-phenyl-3-yridinecarboxylic acid ethyl ester (MRS1477 or short MRS) was synthetized in Dept. of Chemistry, University of Miskolc, Miskolc, Hungary and was dissolved in DMSO at a concentration of 100 mM. 13(S)-hydroxyoctadeca-9Z,11E-dienoic (13(*S*)-HODE) and 8, 9-epoxyeicosatrienoic acids (8, 9-EET), both endogenous agonists of TRPV1 were obtained from Cayman Chemicals (Ann Arbor, MI, USA). Dihydrorhodamine-123 (DHR 123) and Tris–glycine gels were from Molecular Probes (Eugene, OR, USA). Caspase substrates [N-acetyl-Leu-Glu- His-Asp-7-amino-4-methylcoumarin (AC-LEHD-AMC) and N-acetyl-Leu-Glu-His-Asp-7-amino-4-methylcoumarin (AC-LEHD-AMC)] were purchased from Bachem (Bubendorf, Switzerland). A mitochondrial stain 5,5’,6,6’-tetrachloro-1,1’,3,3’-tetraethylbenzimidazolylcarbocyanine iodide (JC-1) was purchased from Santa Cruz (Dallas, TX, USA). Borosilicate capillary glass for patch-clamp experiments was purchased from Sutter Inc. (Novato, CA, USA). All organic solvents were purchased from Santa Cruz (Dallas, TX, USA). The reagents were equilibrated at room temperature for 30 min before any experiments.

### Cell lines and cell extract

MCF7, MDA-MB-231 as well as BT-474 cells of human breast cancer origin (obtained from ATCC) were cultured in RPMI 1640 medium supplemented with fetal bovine serum in a humidified incubator at 37°C, 5% CO_2_ and 95% air. Non-transfected NIH-3T3 and NIH-3T3 murine fibroblast cells permanently expressing rat TRPV1 (NIH-3T3^TRPV1^) were a kind gift from Dr. Zoltan Olah, Institute of Chemistry, University of Miskolc, Hungary. In NIH-3T3^TRPV1^ cells, the metallothionein promoter is used to drive the expression of full-length rat TRPV1 containing additionally a short 12-amino acid ε-tag [21]. These cells express TRPV1 in their plasma membrane at high density and as such they may be used as an indicator cell line for the presence of vanilloids. Cells were cultivated in DMEM containing 10% fetal calf serum and antibiotics (penicillin and streptomycin) at 37°C/5% CO_2_.

Primary breast epithelial cells were isolated from fresh human milk and cultivated according to an established protocol [34]. Milk was diluted 1:1 with phosphate buffered saline (PBS). Milk cells were centrifuged, washed twice with PBS and cultivated in RPMI 1640 culture medium (Gibco Life Technologies, Grand Island, NY, USA), supplemented with 15% fetal bovine serum (Gibco), 10% human serum (Gibco), 1% penicillin-streptomycin (Gibco), 50 ng/ml cholera toxin (Sigma), 0.5 μg/ml hydrocortisone (Sigma), 1 μg/ml insulin (Sigma) at 37°C in a humidified atmosphere (5% CO_2_, 95% air) [35]. Samples were collected, with informed consent and ethical approval. The protocol was approved by the Swiss Cantonal Commission for the Ethics of Human Research No#2016 00309 in accordance with The Swiss Clinical Trial Organisation guidelines.

Approximately 1x10^7^ MCF7 cells were grown in 75 cm^2^ flasks. Cells were washed twice with ice-cold PBS and lysed with lysis buffer. The samples were centrifuged at 4°C for 30 s at 2100 x g. The supernatant was slowly acidified to pH 3.5–4.0 with 0.2 M HCl (25–30 μL acid per each 200 μL sample volume). A 3-fold excess of saturated ethyl acetate was added to the sample and mixed. The sample was centrifuged at 4°C for 5 min at 82 x g to separate the phases and the upper layer (organic phase) was collected. The extraction was repeated three times collecting the organic layers into a single tube. The sample was dried completely and dissolved in 25 μL ethanol. Samples were diluted with buffer solution used for Ca^2+^-imaging experiments (see below) at a 1:1000 dilution. Cell extracts from MCF7, as well as from BT-474 and MDA-MB-231 cells were used for the determination of relative quantities of 13-S-HODE in their lipophilic fraction using an ELISA kit from Enzo Biochem, Inc. (Farmingdale, New York) following the manufacturer’s instruction.

### Ca^2+^ imaging

Cells grown on collagen-coated glass bottom 35-mm dishes (MatTek Corp., Ashland, MA) were loaded with the cell permeable acetoxymethyl (AM)-ester form of the indicator dyes. The following dyes were used: for the cytoplasmic free Ca^2+^ concentration ([Ca^2+^]_i_): Fluo-4-AM (1 μM; Life Technologies, Grand Island, NY) and for the mitochondrial free Ca^2+^ concentration (c_mito_): Rhod-2-AM (1 μM; Life Technologies) diluted in cell culture media for 20 min at room temperature. After loading, cells were washed with buffer solution (DPBS) used for Ca^2+^-imaging experiments that contained (in mM): NaCl 138, Na_2_PO_4_ 8, CaCl_2_ 2, MgCl_2_ 0.5, KCl 2.7, KH_2_PO_4_ 1.6; pH 7.4. The compounds were further diluted with DPBS used for Ca^2+^-imaging experiments. The final concentration of the solvents were <0.1% in all experimental solutions. At these concentrations the solvents did not affect/modify the evoked Ca^2+^ responses in control experiments (data not shown). We used an inverted confocal microscope DMI6000 integrated to a Leica TCS-SP5 workstation to examine changes in [Ca^2+^]_i_. The 488 nm excitation wavelength was used to illuminate the Ca^2+^ indicator Fluo-4. At the confocal microscope, fluorescence emission was recorded at 510–554 nm. Recordings were performed at 37°C using Tempcontrol 37–*2* digital, and a Heating Stage, all from PeCon GmbH (Erbach, Germany). The drugs were added to the abovementioned solutions by pipette and remained in the solution until the end of the experiments. Fluorescence images for [Ca^2+^]_i_ measurements were collected. Circular-shaped regions of interest (ROI) were placed inside the cytoplasmic area of cells. The fluorescence values were calculated after background subtraction (fluorescence intensity of regions without cells). Bleaching correction was carried out, when the baseline was not stable. The relative fluorescent unit (rfu) values were calculated for each cell; fluorescence intensities at each time point were divided by the averaged baseline fluorescence value measured during the non-treatment period. In order to gain insight into evoked Ca^2+^ responses of the entire cell population, the traces of more than 20 randomly selected cells were averaged and standard deviations (SD) were calculated. Each experimental procedure was repeated at least two times with similar results, but only one series of experiments is reported for each case. The LAS-AF (Leica, Wetzlar, Germany) and Prism3 (GraphPad Software, Inc., San Diego, CA) software were used for data analysis.

### Groups for chronic treatment

The MCF7 cells were seeded in 8–10 flasks at a density of 1x10^6^ cells per flask (filter cap, sterile, 250 ml, 75 cm^2^). All cells were cultured at 37°C. The cells were divided into eight groups and all groups let to grow for 72 h in cell culture media containing the following compounds according to the Table 1.

**Table 1:** Treatment groups.

Cell culture medium	+ CAPS (10 μM)	+ CapZ (100 μM)	+ MRS (2 μM)
**Group 1 (control group)**	**-**	**-**	**-**
**Group 2 (CAPS group)**	**+**	**-**	**-**
**Group 3 (CapZ group)**	**-**	**+**	**-**
**Group 4 (CAPS+CapZ group)**	**+**	**+**	**-**
**Group 5 (MRS group)**	**-**	**-**	**+**
**Group 6 (MRS+CAPS group)**	**+**	**-**	**+**
**Group 7 (MRS+CapZ group)**	**-**	**+**	**+**
**Group 8 (MRS+CAPS+CapZ group)**	**+**	**+**	**+**

CAPS, CapZ and MRS were dissolved in DMSO and the pH was adjusted to pH = 7.4 by the addition of HCl. MRS1477 was prepared freshly, but CapZ and CAPS were stored at -33°C. At the end of the treatments, all cells were used for the patch-clamp, apoptosis, cell viability, mitochondrial membrane depolarization, caspase and reactive oxygen species (ROS) assays.

### Electrophysiology

Whole-cell recordings were performed using an EPC 10 amplifier equipped with a personal computer with Patchmaster software (HEKA, Lamprecht, Germany). The standard bath solution contained (in mM): 140 NaCl, 1.2 MgCl_2_, 1.2 CaCl_2_, 5 KCl, 10 HEPES, pH 7.4 (adjusted with KOH). For Na^+^ free solutions, NaCl was replaced by 150 mM N-methyl-D-glucamine (NMDG) and the titration was performed with HCl. The osmolarity of the solution was 310 mosmol/l. The pipette solution contained (in mM) 145 CsCl, 8 NaCl, 2 MgCl_2_, 10 HEPES, pH 7.2 (adjusted with CsOH) and the Ca^2+^ concentration was adjusted to 1 mM (0.886 mM Ca^2+^, 1 mM Cs-EGTA). MCF7 cells were held at a potential of –60 mV and current-voltage (I-V) relationships were obtained from voltage ramps from -90 to +60 mV applied for 400 ms. In the patch-clamp experiment MCF7 cells were perfused with standard bath solution containing CAPS (10 μM) for stimulation or CapZ for inhibition (0.1 mM). The pH of the solutions was adjusted just before starting of the experiments. All experiments were carried out at room temperature (21–23°C). For the analysis the maximal current amplitudes (pA) in a cell were divided by the cell capacitance (pF), a measure of the cell surface. The resulting values represent the current density (pA/pF).

### Cell viability (MTT) assay

To assess MRS’ toxic effects on cell viability, we evaluated the mitochondrial activity of living cells by a 3-(4,5-dimethylthiazol-2-yl)-2,5-diphenyltetrazolium bromide (MTT) quantitative colorimetric assay. After treatment with CAPS and MRS, the cells were washed and incubated with fresh medium containing MTT (0.5 mg/ml) at 37°C for 90 min. Then, the supernatant was discarded and DMSO was added to dissolve the formazan crystals. The absorbance in each well was measured at 650 nm using a microplate reader (Infinite Pro200; Tecan Austria GmbH, Groedig, Austria) [27]. We performed a total of 6 experiments (n = 6) for the cell viability assays. The data were presented as percentage relative to the control.

### Assay for apoptosis markers

Apoptosis was evaluated using the APOPercentage Apoptosis Assay (Biocolor, Belfast, Northern Ireland) according to the manufacturer’s instructions. In a viable cell, maintaining the asymmetric composition of membrane lipids is an energy-dependant process involving the activity of flipase enzymes. The loss of asymmetry serves as an early indicator of apoptosis. When the membrane of an apoptotic cell loses its asymmetry, the APOPercentage dye is actively transported into cells, staining apoptotic cells red, thus allowing detection of apoptosis by spectrophotometry. Absorbance was measured at 550 nm (Infinite Pro200). The data were presented as fold increase normalized to control. The determinations of caspase-3 and caspase-9 activities were based on a method previously reported [36] with minor modifications. Cleavage of the caspase-3 substrate (AC-DEVD-AMC) and caspase-9 substrate (AC-LEHD-AMC) was measured in a microplate reader (Infinite pro200) with an excitation wavelength of 360 nm and emission at 460 nm. The data were calculated as fluorescence units/mg protein and presented as fold increase normalized to control.

### Intracellular ROS measurement

Dihydrorhodamine 123 (DHR 123) is a non-fluorescent, non-charged dye that easily penetrates cell membranes. Once inside the cell, DHR 123 gets fluorescent upon oxidation to yield rhodamine 123 (Rh 123), the fluorescence being proportional to ROS generation. The fluorescence intensity of Rh 123 was measured in a microplate reader (Infinite Pro200). Excitation was set at 488 nm and emission at 543 nm [27]. We performed a total of 6 experiments (n = 6) for the intracellular ROS assays. The data were presented as fold increase normalized to control.

### Mitochondrial membrane potential determination

Cells were incubated with 1 μM JC-1 for 15 min at 37°C as previously described [28]. JC-1 is a lipophilic, cationic dye that can selectively enter into mitochondria and reversibly change color from red to green as the mitochondrial potential decreases. The green signal was measured at an excitation wavelength of 485 nm and an emission wavelength of 535 nm, the red signal at an excitation wavelength of 540 nm and an emission wavelength of 590 nm. Fluorescence values were measured using the microplate reader Infinite Pro200 and the green/red fluorescence intensity ratio was calculated. The data were presented as fold increase normalized to control.

### *In vivo* tumor growth experiment

In accordance with the 3R principles (S1 Text), animal studies were carried out on the basis of data from our *in vitro* experiments indicating that the treatment might be effective also *in vivo*. The sample size calculations were based on the presumption that the treatment would result in a reduction of the average tumor size by at least 1.5 SD. Immunosuppressed NOD SCID gamma (NSG) mice were chosen based on their widespread use as human carcinoma xenograft model [37]. NSG mice, originally obtained from Jackson laboratory, were bred in the SPF animal facility (BRC, Szeged). The health status of the animals was regularly tested by serology and gut content analysis (BioDOC, Hannover, Germany). Animals were kept in an IVC system (Model GA30, Avidin Biotech Ltd, Szeged, Hungary) in an environment enriched with paper tubes at 22±1°C on a 12/12 h light/dark cycle. Four animals per cage were housed in cages of 17 cm x 29 cm surface area. The animals received sterile food and water *ad libitum*. The rodent food pellets and sterile corncob pellet bedding were manufactured by Akronom Ltd (Budapest, Hungary). The animal experiments were performed in accordance with animal experimentation and ethics guidelines of the EU(2010/63/EU). Experimental protocols were approved by the Review Committee of Biological Research Centre Hungarian Academy of Sciences, then by the responsible governmental agency (clearance number: CSI/01/1489-5/2014). Preliminary toxicology experiments not requiring immunosuppressed mice were performed with the commonly used strain C57Bl/6. Female mice (8–10 weeks old, 20–25 g) obtained originally from Jackson laboratory were bred in our animal facility in identical conditions as the NSG mice. For reducing the animal numbers, an “up and down” approach assisted by the AOT425 software was chosen [38]. None of the mice showed any sign of pain or discomfort up to the highest drug dose tested. For the tumor xenograft experiments, young adult (8–10 weeks old) female NSG mice (20–25 g) were randomly assigned to experimental groups, then injected with 2 x 10^6^ MCF7 cells subcutaneously in the flank in 100 μl PBS. It was found previously that MRS show physiological effect administered at dose of 10 mg/kg body weight (b.w.), intraperitoneally. It extended the duration but not the amplitude of capsaicin-evoked hypothermia in mice [26]. Thus, from day 7.5 on, mice were treated with the MRS suspension in a following manner: 10 mg/kg b.w., i.p., twice a week (n = 5 animals per group). The MRS suspension contained as solvent 50% DMSO, 0.5% methylcellulose and 49.5% physiological salt solution. Five control animals received vehicle only. The mice were checked daily for signs of discomfort and tumor growth was followed by palpation twice a week. Mice bearing tumors > 1.5 cm at the largest diameter or mice showing signs of severe pain, discomfort or lethargy are euthanized; in the course of the present experiments, neither the tumors of moderate size, nor the drug treatments caused any visible effect on the well-being (observed as unchanged behavior) of the mice. At the end of the experiment, animals were euthanized by pentobarbital overdose and tumors were dissected and photographed. To exclude subjective bias, tumor measurements were carried out by persons blinded to the treatment groups. The experiments were performed twice with identical results.

### Statistical analysis

Data were analyzed using the Prism (Graphpad) program. All results are expressed as means ± SD. One-way ANOVA (ANalysis Of VAriance) with post-hoc Tukey HSD (Honestly Significant Difference) were performed for the results. P-values of less than 0.05 were regarded as significant.

## Results

### Acute effect of MRS1477 on the [Ca^2+^]_i_

TRPV1 function may be assessed by its ability to pass Ca^2+^ ions across the plasma membrane upon stimulation resulting in changes in [Ca^2+^]_i_. The characteristics of positive allosteric modulators was observed in NIH-3T3 cells stably transfected with TRPV1 channel. This established cell line is highly sensitive to the presence of TRPV1 agonists and therefore may be used as an indicator cell line. MRS augmented the CAPS-evoked rises in [Ca^2+^]_i_ (Fig 1B, gray curve), while MRS, administered alone, did not induce a Ca^2+^ signal (Fig 1C). As a control, the cells were subsequently treated with CAPS that caused a rapid increase in [Ca^2+^]_i_ (Fig 1C). CAPS (50 μM) induced a transient Ca^2+^ signal in primary breast epithelial cells (Fig 1D), while MRS (20 μM) administered alone did not have such an effect on these cells (Fig 1E). A serum-evoked Ca^2+^ response was used as a control to show that cells were viable and moreover capable of a Ca^2+^ response, when stimulated with an appropriate agonist (Fig 1E). CAPS (50 μM), but more importantly also MRS alone (20 μM) evoked a Ca^2+^ response in MCF7 cells (Fig 1F and 1G). We hypothesized that MCF7 cells responded to MRS as the result of the presence of endogenous TRPV1 agonists likely produced by MCF7 cells. To test this hypothesis we carried out a lipid extraction from MCF7 cells and this lipid extract diluted with DPBS (1:1000) was applied to either untransfected (control) or TRPV1-expressing NIH-3T3 cells. Untransfected cells did not respond to either MCF7 cell extracts or to 50 μM CAPS (Fig 1H), while TRPV1-transfected cells showed Ca^2+^ increases in response to both, i.e. lipophilic MCF7 cell extract and CAPS (50 μM) (Fig 1I). These findings confirmed that endogenous TRPV1 agonists were produced by MCF7 cells. Next we tested the MRS effect on the Ca^2+^ response evoked by previously identified endogenous TRPV1 agonists such as 13-HODE (1 μM) and 8,9-EET (1μM). In both cases, MRS augmented the agonist-evoked rises in [Ca^2+^]_i_ (Fig 1J and 1K). Of note, Fluo-4 measurements report on changes in [Ca^2+^]_i_ and not on the Ca^2+^ current passing through the plasmalemmal TRPV1 channels. According to the expectations from a previous study [13], TRPV1 activation in MCF7 cells did not result in a sustained increase in [Ca^2+^]_i_, but rather to a transient Ca^2+^ signal mainly due to the depletion of the endoplasmic reticulum Ca^2+^ stores. In this situation, Ca^2+^ extruding systems were still able to create an equilibrium between the Ca^2+^ influx and Ca^2+^ efflux reverting [Ca^2+^]_i_ close to its basal levels before stimulation. However this new equilibrium requires elevated energy consumption. The exhaustion of Ca^2+^-extruding systems to cope with an excess of Ca^2+^ ions entering the cell through TRPV1 channels may lead to apoptosis even few days later [28]. Thus, we continued our study with the examination of the chronic effects of MRS.

### Effect of PAM pre-treatment on CAPS-induced TRPV1 currents in MCF7 cells

**Fig 2:**
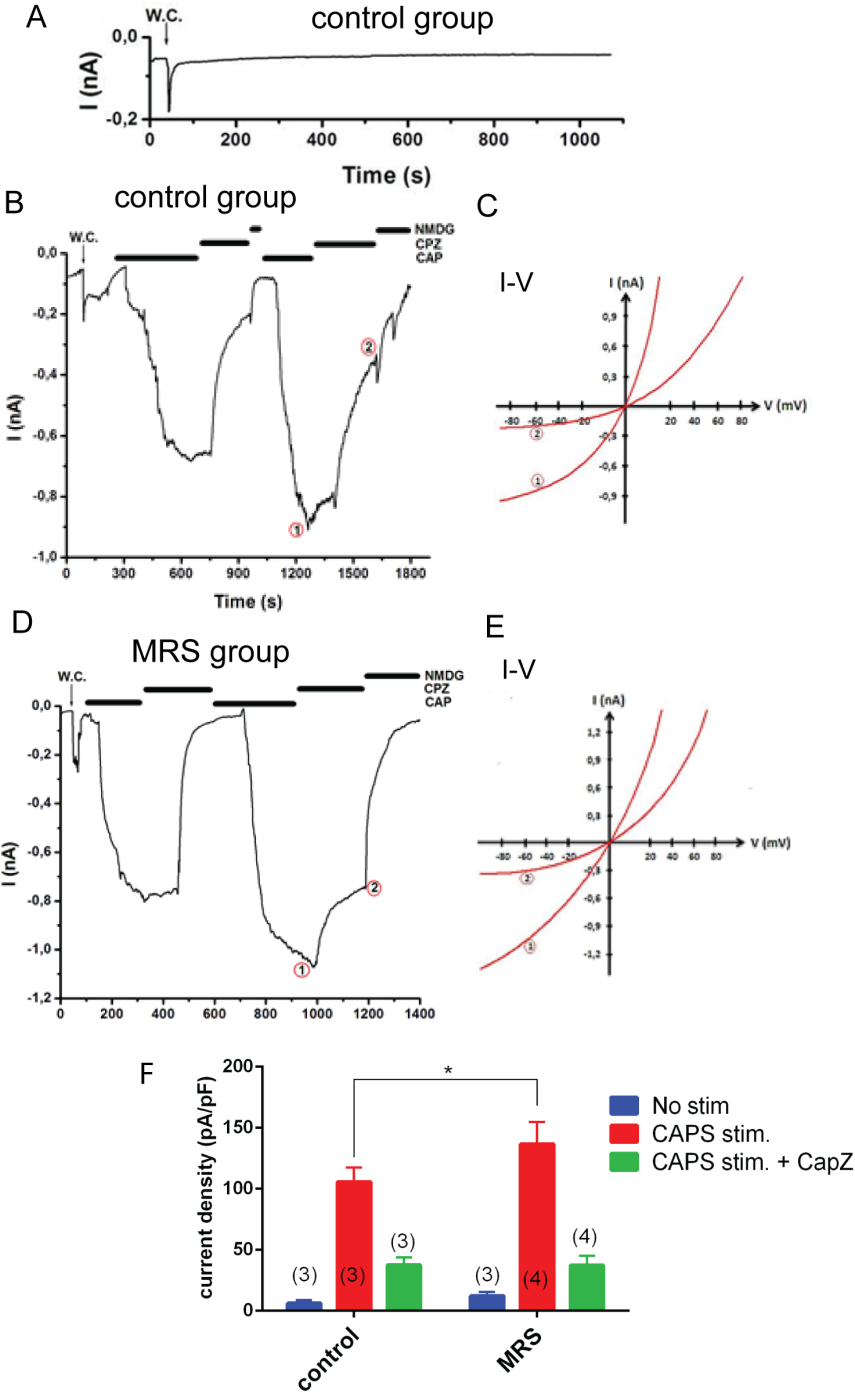
Effects of MRS treatment on TRPV1 channel activity in MCF7 cells. The holding potential was set at -60 mV; W.C. denotes whole-cell patch clamp configuration. **A)** Recording from a control cell without stimulation. **B)** Recording from a cell of the control group: TRPV1 currents in the cells were activated by bath-perfusion of CAPS (10 μM) and subsequently inhibited by the TRPV1 antagonist CapZ (0.1 mM) perfused into the patch chamber. Currents were completely blocked in Na^+^-free solution (NMDG). **C)** Corresponding I/V-relation of currents recorded in **B)** at the indicated time points 1 and 2. **D**) Recording from a cell of the MRS group: cells were incubated with MRS (2 μM for 72 h) prior to the patch-clamp recording. Cells were stimulated by bath-perfusion of CAPS (10 μM) and were inhibited by CapZ (0.1 mM); currents were completely blocked at the end of the experiment by NMDG. **E)** Corresponding I/V-relation of currents recorded in **D)** at the indicated time points. **F)** Current densities were calculated by normalizing the current amplitudes by the cell membrane capacitance. Columns represent the mean + SD. The number of the measurements (n) is indicated within parentheses.

Cells from group 1 (control) and group 5 (MRS-treated) were used for electrophysiological experiments. As mentioned in the method section, holding potentials in the electrophysiological experiments were stable at -60 mV. There was no significant current in unstimulated cells (Fig 2A). The TRPV1 channels in MCF7 cells from the control group were activated by CAPS (10 μM) (Fig 2B). The currents induced by CAPS developed gradually (within 1.82 min) following the addition of CAPS to the medium and reached maximal amplitudes of up to 0.86 nA. We observed reversible and inhibitory effects of CapZ and NMDG^+^ solutions on CAPS-evoked currents (Fig 2B and 2C). The mean values for the current densities (pA/pF) in the Group 1 (control) cells; without CAPS stimulation, with CAPS stimulation and with CapZ treatment after CAPS stimulation were 6 ± 3 (n = 3), 105 ± 12 (n = 3) and 37 ± 7 (n = 3), respectively (Fig 2F). Similar experiments were performed in cells from group 5 (MRS-treated) (Fig 2D and 2E). The mean values for the current densities (pA/pF); without CAPS stimulation, with CAPS stimulation, with CapZ treatment after CAPS stimulation were 12 ± 3 (n = 3), 136 ± 19 (n = 4) and 37 ± 8 (n = 4), respectively (Fig 2F). The comparison of the current density values of group 1 (control) and group 5 (MRS treated) after CAPS stimulation points to a significant effect of MRS pre-treatment on TRPV1 channels (Fig 2F). However, the modulating effect by MRS on CAPS-evoked currents was relatively moderate: (≈30% increase).

### Effect of MRS treatment on the viability of cancer cells

**Fig 3:**
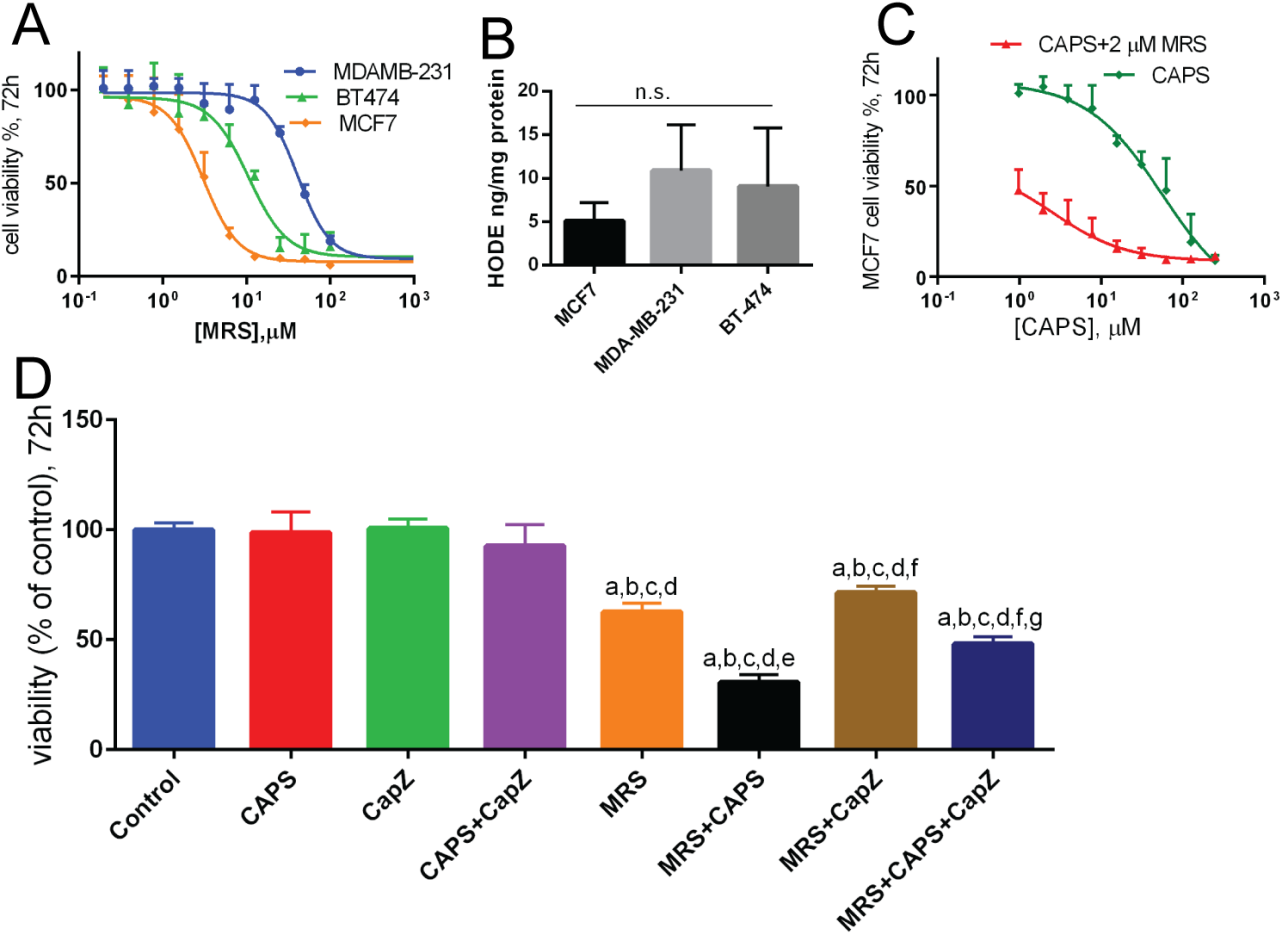
Effects of MRS treatment on cell viability of cancer cell lines. **A.** Cells were treated with MRS at different concentrations for 72 h. At this time point (72 h), MTT assays were performed; each dot represent mean + SD and n = 6 samples (2 independent experiments in triplicates). **B.** HODE levels in the extracts from different cells (n = 3). **C.** Cell viability in the presence of different CAPS concentrations in the absence (green curve) and presence of 2 μM MRS (red curve). MTT assays were performed; each dot represent mean + SD and n = 6 samples (2 independent experiments in triplicates) **D.** Cells were incubated with CAPS (10 μM), CapZ (0.1 mM) or both with and without MRS (2 μM) for 72 h and then MTT assays were performed. The columns represent mean + SD and n = 6 (2 independent experiments in triplicates). The letters denote the following: a—significant difference from control group, b—significant difference from CAPS group, c—significant difference from CapZ group, d—significant difference from CAPS+CapZ group, e—significant difference from MRS group, f—significant difference from MRS+CAPS group and g- significant difference from MRS+CapZ group.

Serial dilutions of MRS were applied to breast cancer cell lines to determine IC_50_ values. The estimated IC_50_ for MRS were the following: 3.0 μM (MCF7), 40.8 μM (MDA-MB-231), 10.6 μM (BT-474) with the following 95% confidence intervals: 2.5–3.7 μM (MCF7), 34.7–47.9 (MDA-MB-231) 8.3–13.6 (BT-474) (Fig 3A). In order to test whether these differences were attributable to the different production rates of 13-HODE in the different cell lines, the relative amount of 13-HODE was determined using ELISA; however no significant differences were found between the cell lines (Fig 3B). Serial dilutions of CAPS were applied to MCF7 cells to determine IC_50_ values of CAPS, a specific TRPV1 agonist, in the presence and the absence of MRS. The determined IC_50_ was 56 ± 12 μM for CAPS without MRS. In the presence of 2 μM MRS the IC_50_ value for CAPS was more than 20-fold decreased to 2.4 ± 0.5 μM indicating a rather strong synergistic effect (Fig 3C). To provide more evidence that a TRPV1-mediated pathway is involved in the toxic effect of MRS, the effects of CAPS, CapZ and MRS on MCF7 cell viability was determined 72 h post-treatment (Fig 3D). While neither CAPS at the chosen concentration nor CapZ alone, nor the combination of the two had an effect on MCF7 viability, MRS (2 μM) significantly decreased (p<0.05) cell viability and it was even further decreased when combining MRS and CAPS (p<0.001). As expected, the viability-decreasing effect of MRS alone could not be rescued by CapZ treatment, since CapZ acts on the extracellular TRPV1 activator site, while MRS acts on the modulatory intracellular PAM site (Fig 1A). In cells treated with MRS and CAPS, co-addition of CapZ slightly increased cell viability; however the viability was still lower than in cells treated with MRS only. This signifies that likely the concentration of CapZ was not sufficient to completely protect from the CAPS-mediated decrease in cell viability (Fig 3D). In summary, this data showed that a TRPV1-mediated pathway is involved in the toxic effect of MRS.

### Effect of MRS treatment on the activation of apoptotic pathways

**Fig 4:**
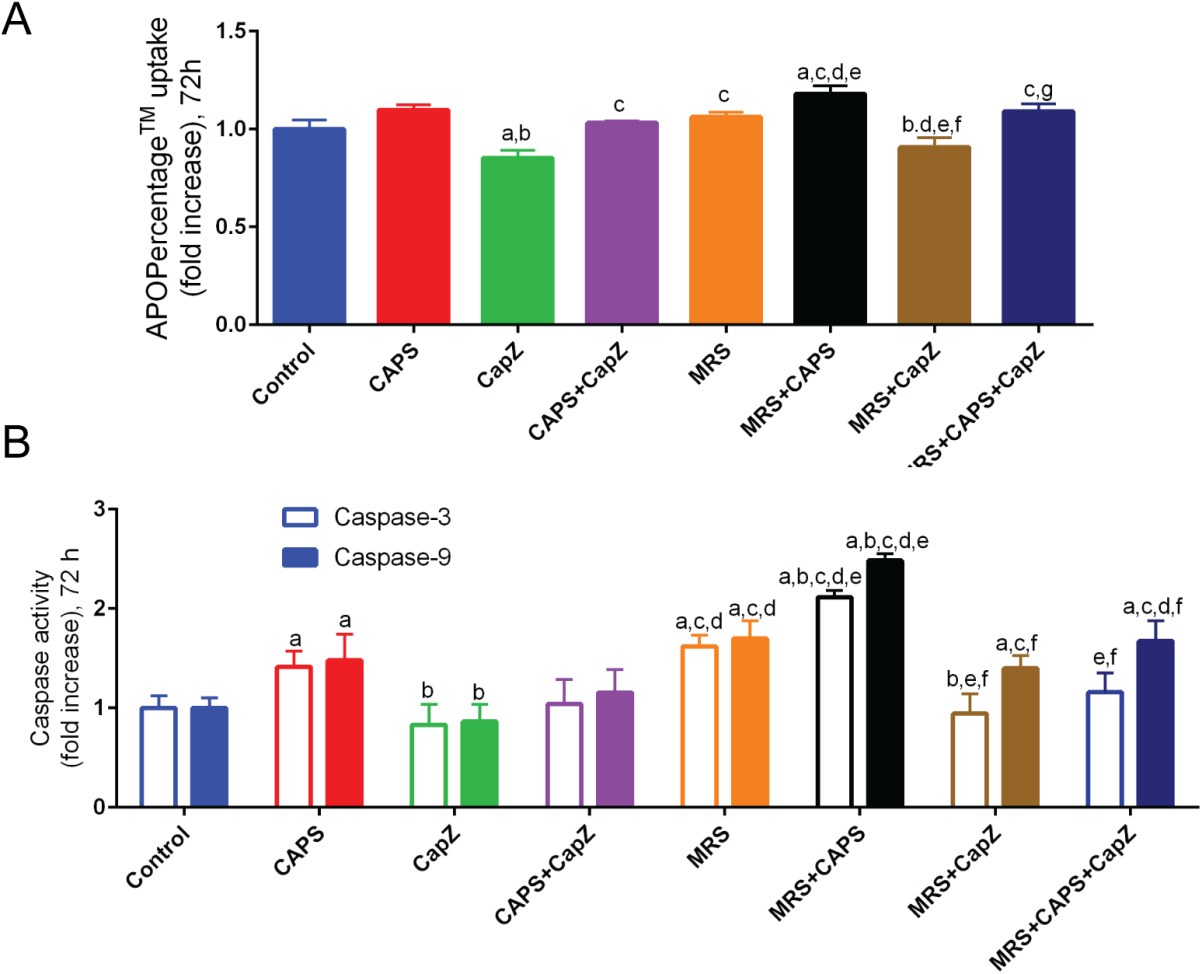
**Effects of MRS treatment on apoptosis evidenced by either loss of lipid asymmetry (A) and caspase-3 and -9 activities (B) in MCF7 breast cancer cells (mean + SD; n = 6, 2 independent experiments in triplicates).** Cells were incubated with CAPS (10 μM), CapZ (0.1 mM) or both with or without MRS (2 μM) for 72 h. Cells were then subjected to the APOPercentage^TM^ assay (indicating the loss of plasma membrane lipid asymmetry) and to caspase activity assays. The letters denote the following: a—significant difference from control group, b—significant difference from CAPS group, c—significant difference from CapZ group, d—significant difference from CAPS+CapZ group, e—significant difference from MRS group, f—significant difference from MRS+CAPS group and g- significant difference from MRS+CapZ group.

The effects of CAPS, CapZ and/or MRS on apoptosis were determined in two independent ways: by determination of the loss of the plasma membrane asymmetry and by measuring the increased caspase activities (Fig 4A and 4B, respectively). Data analyses of the two complementary methods resulted in a rather congruent picture. A loss of membrane asymmetry (higher APOP values) and increased activities of caspase-3 and caspase-9 were observed in the CAPS and MRS groups; values were even higher in the group treated with both, CAPS+MRS. However, normalized values (to control) were also significantly (p<0.05) decreased when CapZ, a TRPV1 inhibitor, was added to the solution during incubation. In addition, all values of the apoptosis markers were further increased in the MRS+CAPS group compared to the CAPS only or MRS only groups (p<0.05). These results indicate an involvement of MRS in the activation of the (likely intrinsic) apoptotic pathway, since activation of caspase-3 (intrinsic) and caspase-9 (intrinsic + extrinsic) pathways were in almost all cases of similar magnitude.

### Effect of MRS treatment on ROS production and mitochondrial membrane depolarization

**Fig 5:**
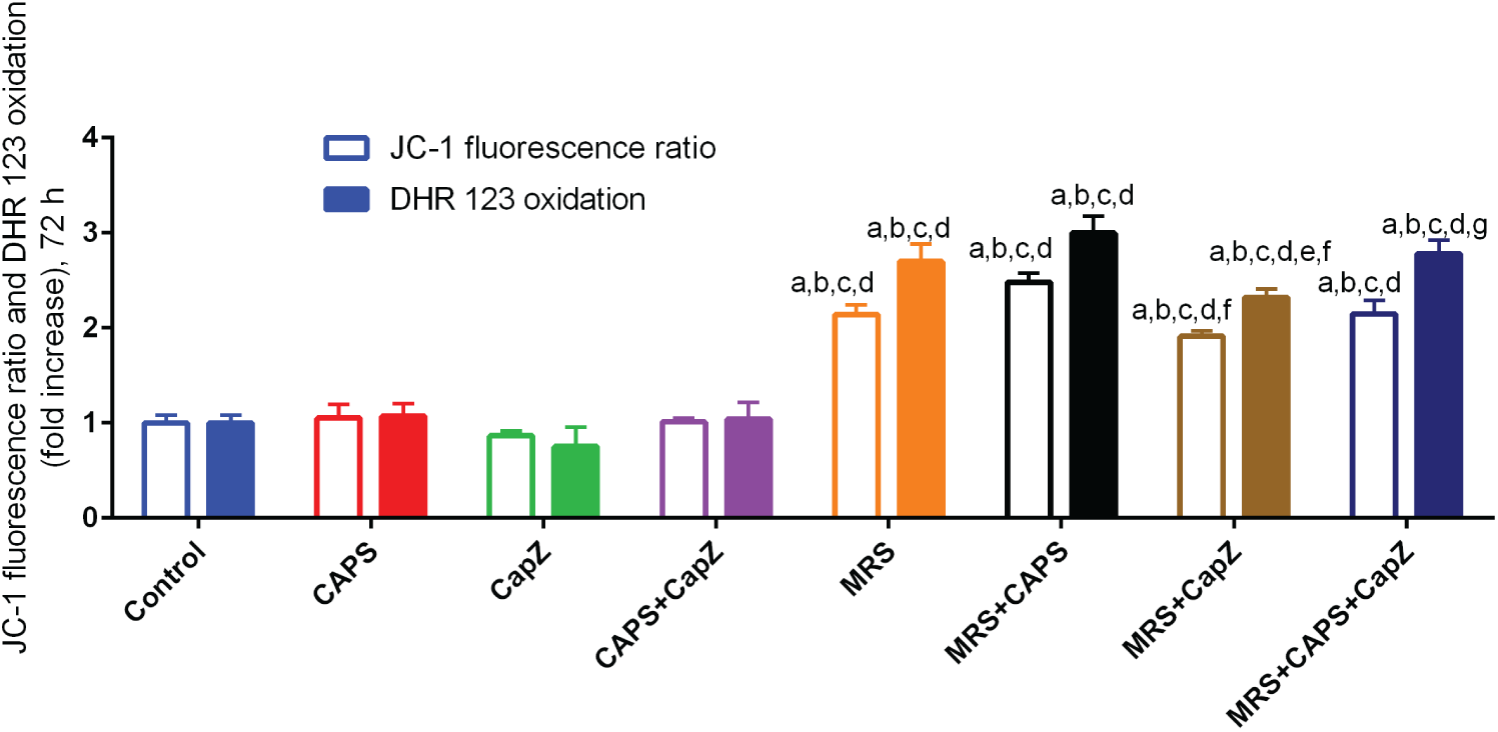
Effects of CAPS and MRS treatment on ROS production and mitochondrial membrane depolarization in MCF7 cancer cells (mean + SD, n = 6, 2 independent experiments in triplicates). Cells were incubated with CAPS (10 μM), CapZ (0.1 mM) or both, with or without MRS (2 μM) for 72 h. Then they were subjected to the DHR 123 and JC-1 assays indicating the levels of ROS production and mitochondrial membrane potential, respectively. The letters denote the following: a—significant difference from control group, b—significant difference from CAPS group, c—significant difference from CapZ group, d—significant difference from CAPS+CapZ group, e—significant difference from MRS group, f—significant difference from MRS+CAPS group and g- significant difference from MRS+CapZ group.

Most Ca^2+^ ions entering a cell caused by hyper-activity of TRPV1 channels are taken up into mitochondria [13]. Subsequently, mitochondrial Ca^2+^ accumulation leads to mitochondrial depolarization, i.e. the mitochondrial membrane potential (Ψ_m_) diminishes resulting in an increase in intracellular ROS production and oxidative stress [39]. Mitochondrial membrane depolarization (estimated by the JC-1 assay and reported as a ratio) and ROS production (estimated by the DHR assay) were unaffected by CAPS alone; accordingly, the addition of CapZ or the combination of both had also no effect on mitochondrial membrane potential and ROS production (Fig 5). On the contrary, in all groups subjected to a MRS treatment (MRS, MRS+CAPS, MRS+CapZ, MRS+CAPS+CapZ) values for both measurements were significantly increased (p<0.001) compared to the control group. The essentially unchanged values for MRS compared to MRS + CAPS indicated that the effect was mostly mediated by MRS. Addition of CapZ (MRS + CapZ group), assumed to essentially block the effects of CAPS, partially restored (decreased) the values for mitochondrial membrane depolarization and ROS production (p<0.05) compared to the MRS+CAPS group. In addition, these values were also higher in the MRS+CAPS group compared to the CAPS only group (p<0.001; for more details, see Fig 5). These results point towards a significant effect of MRS on mitochondrial activity and oxidative stress. Moreover, it supports the presence of an endogenous activator of TRPV1 channels produced by MCF7 cells that is strongly modulated by MRS, but most likely not via the CAPS-binding site.

### *In vivo* treatment of MCF7 tumors with MRS

**Fig 6:**
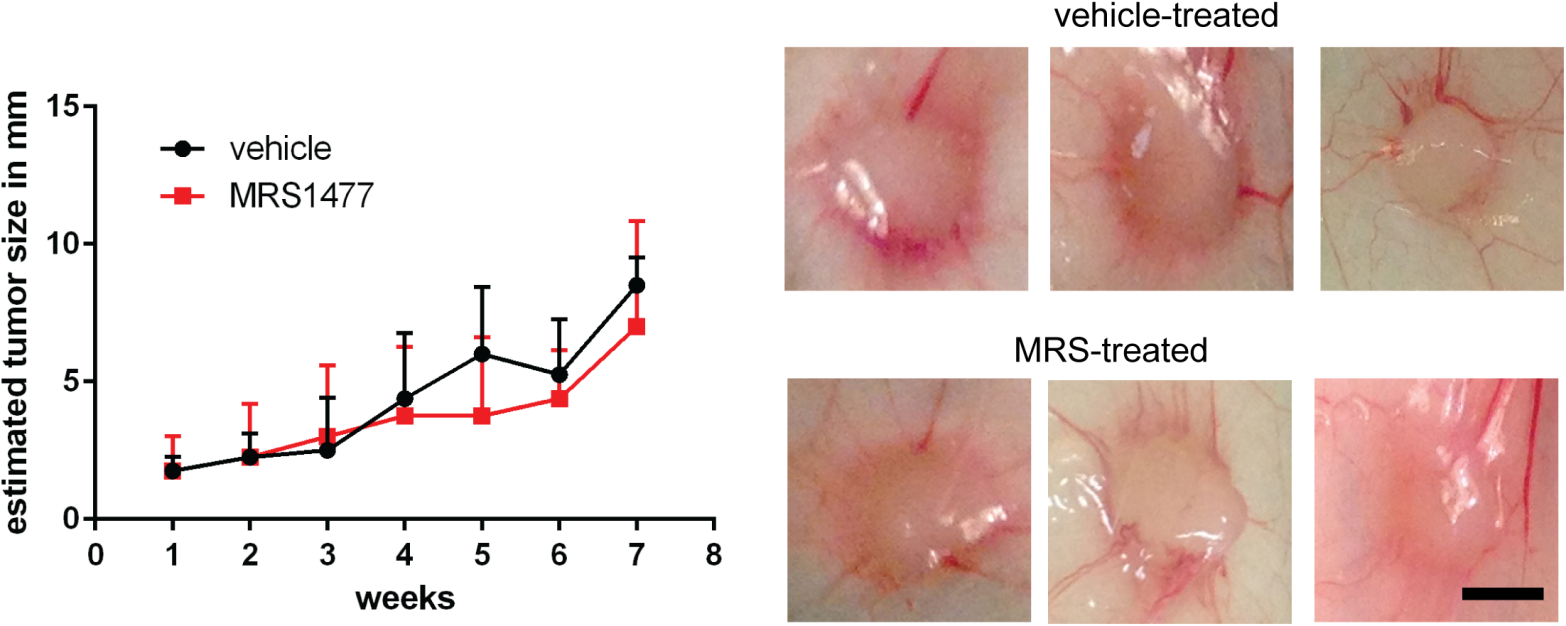
*In vivo* effect of MRS in NSG mice bearing MCF7 cell-derived tumors. **A)** No significant difference in tumor size was observed between MRS-treated (10 mg/kg body weight (b.w.), i.p., twice a week) and vehicle-treated groups. Student t-tests were used for pairwise comparisons. Bars represent SD. **B)** Photographs taken from tumors derived from vehicle- and MRS-treated mice. No obvious structural differences were observed at the macroscopic level. Size bar represents 5 mm.

Based on the rather strong effects of MRS on MCF7 cells *in vitro*, i.e. a decrease in cell viability, an increase in apoptosis and larger production of ROS, we assessed the putative antitumor capacity of MRS in a mouse model *in vivo*. C57Bl/6J mice (n = 5 animals) were treated with MRS (100 mg/kg body weight) intraperitoneally, twice per week, in order to check for general *in vivo* toxicity; no apparent toxic effects were observed (e.g. no behavioral alterations, signs of pain (fur, posture) or animal death). In a following experiment, NSG mice (n = 5 animals per group) were injected with MCF7 cells (2 x 10^6^ cells; s.c). After 7.5 days of unrestricted tumor growth, one group was treated with MRS (10 mg/kg body weight;, i.p., twice per week), the other group was treated with vehicle (saline). The tumor growth was estimated by caliper measurements in both groups during a total of 8 weeks after MRS treatment. No significant differences in tumor size were observed between the two groups (Fig 6A). Also the macroscopic appearance of the tumors (shape, color, vascularization) was not different in the sham-treated *vs*. MRS-treated mice (Fig 6B).

## Discussion

Activation of TRPV1 channels with endo- and exovanilloids leads to pain sensation; moreover excessive and/or prolonged activation may lead to axonal destruction or even cell death of nociceptive neurons and may evoke various biological responses in non-neural cell types [5,13,33,40]. Endogenous TRPV1 ligands have likely different pharmacological properties (e.g. affinity, potency, metabolic rate, etc.) compared to naturally occurring exogenous agonists such as CAPS or RTX and consequently endogenous ligands might have different physiological functions. As an example, endogenous agonists are involved in the generation of chronic pain, while exogenous agonists are capable of alleviating chronic pain due to the process of desensitization [41]. Similarly, although the tumor environment was shown to contain endogenous TRPV1 agonists promoting the development of tumors [42], exogenous agonists such as CAPS might have an opposite, i.e. anti-tumorigenic effect. Beneficial effects of CAPS for the treatment of prostate cancer *in vivo* have been reported before. A prostate cancer patient, who ingested chili sauce twice a week maintained a stable prostate-specific antigen (PSA) reading for a year [43]; PSA is an established marker for the presence and activity of prostate tumors. An oral dose of CAPS (5mg/kg b.w./day) significantly slowed down the growth of PC-3 prostate cancer xenografts in *in vivo* mouse models [44,45]. A large cohort study revealed that consumption of spicy food is inversely correlated with the mortality caused by cancer, ischemic heart diseases and respiratory diseases [46]. The mechanism of how tumor cells are influenced by CAPS consumption is not entirely known. The two proposed mechanisms include I) that CAPS might interfere with the endogenous weaker agonists present in the tumor milieu or II) that CAPS influences the tumor innervation by sensory neurons.

Upregulation of TRPV1 channels in neoplastic breast and prostate tissue compared to normal tissue has been reported before [47,48], but tumors express lower levels of TRPV1 than sensory neurons [13]. Results from our experiments indicate that MCF7 cells not only express functional TRPV1 channels, but also produce endogenous TRPV1 ligands based on our findings that I) in MCF7 cells, MRS caused a transient increase in [Ca^2+^]_i_, while not in the other cell lines tested. II) In a previous study we had found that in MCF7 cells merely the overexpression of TRPV1 channels decreased the viability of cancer cells [13]. We observed that mainly apoptotic processes were activated, but also mitotic arrest in MCF7^GFP-TRPV1^ cells was detected. The absence of mitosis in the surviving MCF7^GFP-TRPV1^ cells subsequently didn’t allow for the establishment of stable MCF7^GFP-TRPV1^ clones, although we had been successful to establish cell clones permanently expressing ectopic TRPV1 protein using non-tumor derived cell lines such as HaCaT, a spontaneously immortalized keratinocyte cell line from adult human skin [33], or NIH-3T3 cells, a spontaneously immortalized mouse embryo fibroblast cell line [49]. Nevertheless, this phenomenon might be explained by the production of endogenous TRPV1 agonists, constantly activating the ectopically expressed TRPV1 channels in MCF7 cells.

Here, we showed that a lipophilic extract from MCF7 cells must contain at least one TRPV1 agonist, because one (or several) components of the lipophilic extract was capable of activating the TRPV1 receptor. The presence of 13-HODE was identified (Fig 3C) but certainly other known or yet unknown endogenous TRPV1 agonists [50] are likely to be present in the lipophilic extract, whose molecular identification will require further studies. Nevertheless some predictions can be made based on previous studies. The oxidized linoleic acid metabolite 13-S-hydroxyoctadecadienoic acid (13-HODE) is present in human prostate adenocarcinoma specimens, while it is absent in adjacent normal tissue [51]. Also a low pH in the extracellular milieu activates TRPV1 [8,52] and is associated with inflammation and the cancer microenvironment [53,54]. LNCaP prostate cancer cells produce high amounts of 20-hydroxyeicosatetraeonic acid (20-HETE) [55], another identified endogenous activator of TRPV1 channels [56]. Expression of 12-lipoxygenase, the enzyme involved in generation of 12(*S*)-HETE, is correlated with more malignant stages of prostate cancer [57,58]; 12(*S*)-HETE is a well-known endo-agonist ligand of TRPV1, shown to increase the firing rate of sensory neurons *via* activation of the TRPV1 channel opening [59].

In our study, we applied the positive allosteric modulator (PAM) concept knowing that PAM alone, in our case MRS, does not have an intrinsic activity on TRPV1 channels [26] unlike exogenous vanilloid agonists or antagonists. MRS does not induce pain similar to TRPV1 agonists acting on pain sensing neurons [25]. The action of PAMs, such as MRS and MRS-analogs, are restricted to the site of the production of endogenous agonists of TRPV1; i.e. most probably specific to the inflammatory milieu of the proximal tumor environment. Here we demonstrated that MRS does not interfere directly with TRPV1 function, e.g. as shown by the absence of any increase in [Ca^2+^]_i_ in TRPV1-expressing NIH-3T3 cells (Fig 1C). Only in the presence of not-identified endogenous agonist(s) produced by MCF7 cells, MRS caused transient increase in [Ca^2+^]_i_. Based on these results, we conclude that vanilloids and PAM added together, might act in a synergistic manner by inducing oxidative stress and increasing apoptosis, finally resulting in a remarkable decrease in MCF7 cell viability *in vitro*. If the [Ca^2+^]_i_ influx increases, the exhausting energy-consuming Ca^2+^-extruding systems might be unable to cope with it. This in turn leads to intracellular ROS production and mitochondrial membrane depolarization [27]. Mounting evidence indicates that production of excessive ROS and stimulation of apoptotic pathways, including caspase-3 and -9 activities, are increased in MCF7 cells by increased mitochondrial membrane depolarization [28,60]. Our results support the hypothesis that CAPS- and MRS-induced elevated Ca^2+^ influx leads to mitochondrial membrane depolarization, excessive ROS production and apoptosis. Although a previous study had shown that MRS is a specific and cognate modulator of TRPV1 channels [24], the fact that a relatively modest (30%) increase in Ca^2+^ influx results in a huge change of mitochondrial functions raises the possibility that MRS has an off-target effect on a mitochondrial protein involved in mitochondrial Ca^2+^ regulation.

In our *in vivo* study, however, we have not tested whether co-application of e.g. CAPS together with MRS would act synergistically and promote anti-tumor activity. We assumed that endogenous TRPV1 activators together with MRS might be sufficient to impair MCF7 tumor formation. It appears that the used concentration and/or the pharmacokinetic properties of MRS compound are not appropriate to impair tumor growth *in vivo* (Fig 6).

In conclusion, this study’s results indicated that MRS1477 alone or together with CAPS has an apoptotic effect on MCF7 breast cancer cells in *in vitro* assays. This might be related to up-regulation of Ca^2+^ entry, intracellular ROS production, caspase-3 and caspase-9 activities. Although administered alone MRS1477 did not show significant antitumor activity, it still might be useful in combination with low dose of CAPS or with cytostatic compounds such as cisplatin. Future experiments should aim to find a combination of CAPS and various PAMs that might result in tumor regression *in vivo* without detrimental effects on the normal non-transformed cells also expressing TRPV1. Thus, despite of the promising *in vitro* results, further optimizations for a putative cancer treatment *in vivo* are required.

## Supporting information

S1 Text This document contains the NC3Rs ARRIVE guidelines checklist-filled. (PDF)
